# Annual Report of the Surgeon-General U. S. A.

**Published:** 1863-01

**Authors:** William A. Hammond, E. M. Stanton

**Affiliations:** Surgeon-General; Sec’y of War


### ANNUAL REPORT OF THE SURGEON-GENERAL U. S. A.

*Surgeon-General's Office, Nov.* 10, 1862.

Sir:—I have the honor to lay before you a statement of the fiscal transactions, and a report upon the operations generally, of the Medical Department of the Army, for the fiscal year ending on the 30th of June, 1862.

The amount of the appropriation for the Medical and Hospital Department on the 30th of June, was:

| | | |
|---|---|---|
| In the hands of disbursing agents,........ | | $6,006.62 |
| In the Treasury of the United States,...... | | 41,172.92 |
| Amount appropriated per Act July 17, 1861, | | 1,271,841.00 |
| Amount appropriated per Act Feb. 25, 1862, | | 1.000,000.00 |
| Amount appropriated for deficiency to June, 30, '62, approved Feb. 25, 1862,....... | | 125,000.00 |
| Amount refunded into the Treasury, on acc't Medical and Hospital stores sold at auction, viz, D. D. Morrison $330.60, John Moore $950.50, E. H. Abadie $330.43, I. D. Cotton $240.00, Samuel Elliott $18.32,..... | | 1,874.35 |
| Total,.... | | 2,445,894 89 |
| Of this sum there has been expended on account of pay, etc. of private physicians, contracted in 1861,.................. | 35,052.91 | |
| do do 1862,.................. | 86,597.76 | |
| For medicines, instruments, hospital stores, etc. ..... ........................ | 2,249,472.52 | 2,371,113.18 |
| Leaving in the hands of disbursing agents, | | 74,781.70 |

It has been usual for a report of the sickness and mortality of the Army to accompany this report, but it is found impracticable, arising from the vast amount of labor incident thereto, and it will be furnished, it is believed, in time for publication as a supplement to the "Surgeon-General's Report for the fiscal year ending June 30, 1862."

The number of General Hospitals is 150, and the total number of patients in them, $58.715.

During the past year the health of the troops has been remarkably excellent. No epidemics of any severity have appeared among them, and those diseases which affect men in camp have been kept at a low minimum.—Scurvy has been almost entirely prevented, and yellow fever, from which much was feared, has had but few victims. This immunity is due to the excellent hygienic arrangements instituted, and to the cordial manner in which Generals in command have co-operated with the proper authorities,

In an army of the size of that now maintained by the United States, it was of course to be expected that the absolute number of sick would be very large, and the important battles which have been fought have thrown a large number of wounded on the care of the Department. At present the total number under the charge of officers of the Medical Department is not short of 70,000, and immediately after the battle of Antietam it was over 90,000. That this large number could be provided for without some cases of unnecessary suffering occurring, would perhaps be too much to expect; but I must commend the Medical Corps, both of the Regular and Volunteer service, for the faithful and efficient manner in which their duties have been performed. In the discharge of their duties Medical Officers have been very much aided by the contributions of the people of the country, and by the efficient co-operation of the Sanitary Commission and Relief Associations.

In addition to providing the sick and wounded with medical attendance and medicines, much has been done by the Department in furnishing food, clothing, and comforts of various kinds. From much observation, both at home and abroad, and from the concurrent testimony of distinguished foreign medical officers, I am satisfied that never before were the sick and wounded of an army so well cared for as are those who have suffered for their country in the present rebellion. The hospitals, I take pride in saying, are a credit to the nation.

Before the several medical boards in session during the year (from July 1st, 1861, to June 30th, 1862,) a large number of applicants for appointment in the medical staff of the Army were invited by the Secretary of War. Of these sixty-six candidates duly presented themselves. Thirty-three of this number were approved, and five rejected; the remaining twenty-eight withdrew, one on account of physical disqualification. Before the same Boards eleven Assistant Surgeons were examined for promotion, nine of whom were found qualified, and two not considered as coming up to the standard of merit required. In the examination by these Boards, the standard of attainments required for success was much lowered, the Board in New York being ordered to examine two candidates each day for the regular army, while the examination of candidates for the appointment of Surgeon of Brigade became little more than a farce. Since the 1st of June last, however, the standard of examination has been raised, and the gentlemen now entering the Medical Staff have been found fully competent to undertake the important trust with which they are charged.

The breaking out of the rebellion found the United States Army with a Medical Department arranged for a peace establishment of 15,000 men. Experience soon demonstrated the fact, that, however efficient its officers might be, the organization was such as to ill adapt it to the necessities of a large force in time of war. Partial progress in the right direction was made by Congress in increasing the rank of the Surgeon-General, adding a limited Inspecting Corps, and increasing the number of Surgeons, Assistant Surgeons, Medical Cadets, and Hospital Stewards. The Department was also placed on a more independent footing, and its whole status elevated. But there are still other measures, which, if adopted, cannot fail to add to the efficiency of the Department, and these I desire to urge through you on the attention of Congress.

First among these is the establishment of a permanent Hospital and Ambulance Corps, composed of men specially enlisted for duty in the Medical Department, and properly officered, who shall be required to perform the duties of nurses in the hospitals, and to attend to the service of the ambulances in the field. By the establishment of this corps several thousand soldiers, now detached as nurses, cooks, etc., would be returned to duty with their regiments, and the expense now incurred by the necessary employment of contract nurses obviated. A corps formed upon the basis of two men to each company in service, organized into companies of 100 privates, with one Captain, two Lieutenants, four Sergeants, and eight Corporals to each Company, would relieve the line of the Army from all details for the Medical Department, and enable the Department to render far more efficient services to the sick and wounded than it is capable of affording under the present system. The necessity of such a corps has been recognized in all European armies, and I am able to speak from personal observation of the great advantages to be derived from it.

I regard an increase of the Medical Corps, both of the regular and volunteer forces, as absolutely necessary. The law of Congress, approved July 2d, 1862, provides sufficiently, except for Cavalry and Artillery regiments, for the wants of troops in the field, but the service in hospitals has to be filled to a great extent by the employment of contract physicians. I therefore recommend that the Medical Corps of the Regular Army be increased by twenty Surgeons and forty Assistant Surgeons, and the Staff Corps of Volunteer Medical Officers by fifty Surgeons and two hundred and fifty Assistant Surgeons. This last Corps now consists of 200 Surgeons and 120 Assistant Surgeons. The Cavalry and Artillery organization requires Medical Officers as much as Infantry. The omission on the part of Congress should be supplied; a Surgeon and two Assistant Surgeons should be authorized for each regiment of Cavalry, and for each regiment of heavy artillery, and an Assistant Surgeon to each light Battery.

Under the first section of the Act of June 30th, 1834, Assistant Surgeons of the regular army must have served five years before being eligible for promotion as Surgeon. On the 1st of November there were but six Assistant Surgeons in the army who had served five years. The effect of this law will be to prevent the filling of vacancies which may occur in the grade of Surgeon, and I therefore recommend that so much of said section as requires Assistant Surgeons to serve five years as such, before being eligible surgencies, be repealed.

The number of Medical Cadets is altogether too small for the necessities of the service. I therefore recommend that authority be given to appoint as many as may be required, in accordance with existing laws on the subject.

The institution of a Medical Inspecting Corps has been productive of excellent results. The number of Inspectors authorized is, however, too limited to enable the service to be as efficiently performed as is desirable. I therefore recommend that two Inspectors General and eight Inspectors be added to the present organization. The authorization of an additional Assistant Surgeon-General would also be a measure of great propriety.

Considerable progress has been made in the establishment of an Army Medical Museum. The advantages to the service and to science from such an institution cannot be over estimated. I respectfully recommend that a small annual appropriation be made for its benefit.

An Army Medical School, in which Medical Cadets and others seeking admission into the Corps, could receive such special instruction as would better fit them for commissions, and which they cannot obtain in the ordinary medical schools, is a great desideratum. Such an institution could be established in connexion with any General Hospital, with but little if any expense to the United States. A hospital of a more permanent character than any now in this city is, I think, necessary, and will be required for years after the present rebellion has ceased. I therefore recommend that suitable buildings be purchased or erected for that purpose. If this is done the Medical School and Museum will be important accessions to it.

Experience has shown that a most useful class of officers was authorized by the Act relative to Medical Storekeepers. The number now authorized is too small. They could very properly perform the duties of medical purveyors, now performed by medical officers, and thus officers who have been educated with special reference to service as physicians and surgeons, and who are now acting as medical purveyors, would be enabled to resume their proper duties. I therefore recommend an addition to the medical storekeepers.

At present the washing of clothes in General Hospitals is provided for as follows: One matron is provided for every twenty patients, who receives a compensation of six dollars per month and one ration. Great difficulty is experienced in large General Hospitals in procuring a sufficient number of matrons to peform this duty, and I have the honor to propose that, instead of this now unreliable plan, a sum of money, equivalent to the pay and allowance of a matron, say twelve dollars for every twenty patients, be monthly allowed to every General Hospital, to be appropriated for laundry purposes at the discretion of the Surgeon in charge, whether to the payment of matrons or the payment of bills for washing by steam or otherwise.

The tenth section of the Act approved July 17, 1862, gives additional rank to officers of the Adjutant General's, Quarter Master's, Subsistence and Inspector General's Department, who are serving on the Staff of Commanders of Army Corps. There is, I think, manifest propriety in extending the provisions of this Act to the officers of the medical department who may be on duty with such command as medical directors, and I respectfully ask for such extension.

The Engineer and Ordnance Departments are charged with the erection of buildings which requires special knowledge. The building of hospitals also requires knowledge of a peculiar character, which is not ordinarily possessed by officers out of the medical department. It would therefore appear obviously proper that the medical department should be charged with the duty of building the hospitals which it is their duty to administer.

In the matter of transportation the interests of the service require that the medical department should be independent. Much suffering has been caused by the impossibility of furnishing supplies to the wounded, when those supplies were within a few miles of them in great abundance.

The establishment of a laboratory, from which the medical department could draw its supplies of chemical and pharmaceutical preparations, similar to that now so successfully carried on by the medical department of the Navy, would be a measure of great utility and economy. I therefore respectfully recommend that authority be given for this purpose.

In regard to the age at which recruits are received into service a change is imperatively demanded, both for the interest of the Army and the welfare of individuals. The minimum is now fixed at eighteen years, and it is not uncommon to find soldiers of sixteen years old. Youths of these ages are not developed, and are not fit to endure the fatigues and deprivations of military life. They soon break down, become sick, and are thrown upon the hospitals. As a measure of economy I recommend that the service age of recruits be fixed by law at twenty years.

The present manner of supporting the cartridge-box is productive of hernia or rupture. Many instances in support of this statement have occurred since the commencement of the rebellion, and reports on the subject are frequently received from medical officers. I recommend that, instead of being carried by a belt around the waist, the cartridge-box be supported by a shoulder-strap. This would entirely obviate the evil.

At the last session of Congress the sum of two millions of dollars was appropriated for the relief of discharged soldiers. I recommend that one million of dollars of this sum be set aside for the establishment of a permanent home for those who have been disabled in their country's service. This measure is one of such importance that I forbear entering into details at this early period. An establishment of the kind organized upon an approved plan would be productive of incalculable benefit.

Soon after my appointment I issued circulars to medical officers, inviting them to co-operate in furnishing materials for a Medical and Surgical History of the Rebellion. A large number of memoirs and reports of great interest to medical science, and military surgery especially, have been collected, and are now being systematically arranged. The greatest interest is felt in this labor by the medical officers of the Army and physicians at large.

The reorganization of the Medical Department necessitated a new set of regulations for its guidance. Under your orders a Board has been in session preparing a new code. Their labors have been very much interfered with by the necessity of detailing them, from time to time, for more imperative duties, but I expect to be able to submit to you, in a short time, a complete set of regulations for your approval.

I have deemed it my duty, with your sanction, to visit, from time to time, the hospitals and armies of the eastern portion of the country. I have thus been enabled to make myself acquainted with their sanitary condition and medical wants. I hope, ere long, to be able to extend these inspections to the west.

A uniform diet table for General Hospitals has been prepared with great care, and promises to work advantageously.

Large deposits of medical supplies have been established at New York, Philadelphia, Baltimore, Fortress Monroe, Washington, Cincinnati, Cairo, St. Louis, and Nashville, which have proved of incalculable advantage to the sick and wounded. Moreover, large sums have been saved by the accumulation of stores before the recent advance took place.

In terminating my report, I desire to express the hope that the labors of the the Officers of the Medical Department may be made more and more worthy of the high mission which has been confided to them,

I am, Sir, very respectfully, your obedient servant,

William A. Hammond,
Surgeon-General.

Hon. E. M Stanton, Sec'y of War.

